# Applying Mendelian randomization to appraise causality in relationships between nutrition and cancer

**DOI:** 10.1007/s10552-022-01562-1

**Published:** 2022-03-11

**Authors:** Kaitlin H. Wade, James Yarmolinsky, Edward Giovannucci, Sarah J. Lewis, Iona Y. Millwood, Marcus R. Munafò, Fleur Meddens, Kimberley Burrows, Joshua A. Bell, Neil M. Davies, Daniela Mariosa, Noora Kanerva, Emma E. Vincent, Karl Smith-Byrne, Florence Guida, Marc J. Gunter, Eleanor Sanderson, Frank Dudbridge, Stephen Burgess, Marilyn C. Cornelis, Tom G. Richardson, Maria Carolina Borges, Jack Bowden, Gibran Hemani, Yoonsu Cho, Wes Spiller, Rebecca C. Richmond, Alice R. Carter, Ryan Langdon, Deborah A. Lawlor, Robin G. Walters, Karani Santhanakrishnan Vimaleswaran, Annie Anderson, Meda R. Sandu, Kate Tilling, George Davey Smith, Richard M. Martin, Caroline L. Relton, with the M. R. in Nutrition, Cancer working group

**Affiliations:** 1 Population Health Sciences, Bristol Medical School, University of Bristol, Bristol, UK; 2 Medical Research Council (MRC) Integrative Epidemiology Unit (IEU) at the University of Bristol, Bristol, UK; 3 Departments of Nutrition and Epidemiology, Harvard TH Chan School of Public Health, Boston, MA USA; 4 Bristol National Institute for Health Research (NIHR) Biomedical Research Centre, Bristol, UK; 5 Clinical Trial Service Unit and Epidemiological Studies Unit (CTSU) and the Medical Research Council Population Health Research Unit (MRC PHRU), Nuffield Department of Population Health, University of Oxford, Oxford, UK; 6 School of Psychological Science, University of Bristol, Bristol, UK; 7 Department of Economics, Vrije Universiteit Amsterdam, Amsterdam, The Netherlands; 8 Department of Applied Economics, Erasmus School of Economics, Erasmus University Rotterdam, Rotterdam, The Netherlands; 9 K.G. Jebsen Center for Genetic Epidemiology, Department of Public Health and Nursing, NTNU, Norwegian University of Science and Technology, Trondheim, Norway; 10 International Agency for Research On Cancer (IARC), Lyon, France; 11 Nightingale Health Oy, Helsinki, Finland; 12 Cellular and Molecular Medicine, Faculty of Life Sciences, University of Bristol, Bristol, UK; 13 Department of Health Sciences, University of Leicester, Leicester, UK; 14 MRC Biostatistics Unit, University of Cambridge, Cambridge, UK; 15 Department of Public Health and Primary Care, University of Cambridge, Cambridge, UK; 16 Northwestern University Feinberg School of Medicine, Chicago, IL USA; 17 Research Innovation Learning and Development (RILD) Building, University of Exeter Medical School, Exeter, UK; 18 Hugh Sinclair Unit of Human Nutrition, Department of Food and Nutritional Sciences, University of Reading, Reading, UK; 19 Population Health and Genomics, School of Medicine, University of Dundee, Dundee, Scotland, UK; 20 NIHR Biomedical Research Centre, Bristol, UK

**Keywords:** Cancer, Causality, Nutrition, Mendelian randomization

## Abstract

Dietary factors are assumed to play an important role in cancer risk, apparent in consensus recommendations for cancer prevention that promote nutritional changes. However, the evidence in this field has been generated predominantly through observational studies, which may result in biased effect estimates because of confounding, exposure misclassification, and reverse causality. With major geographical differences and rapid changes in cancer incidence over time, it is crucial to establish which of the observational associations reflect causality and to identify novel risk factors as these may be modified to prevent the onset of cancer and reduce its progression. Mendelian randomization (MR) uses the special properties of germline genetic variation to strengthen causal inference regarding potentially modifiable exposures and disease risk. MR can be implemented through instrumental variable (IV) analysis and, when robustly performed, is generally less prone to confounding, reverse causation and measurement error than conventional observational methods and has different sources of bias (discussed in detail below). It is increasingly used to facilitate causal inference in epidemiology and provides an opportunity to explore the effects of nutritional exposures on cancer incidence and progression in a cost-effective and timely manner. Here, we introduce the concept of MR and discuss its current application in understanding the impact of nutritional factors (e.g., any measure of diet and nutritional intake, circulating biomarkers, patterns, preference or behaviour) on cancer aetiology and, thus, opportunities for MR to contribute to the development of nutritional recommendations and policies for cancer prevention. We provide applied examples of MR studies examining the role of nutritional factors in cancer to illustrate how this method can be used to help prioritise or deprioritise the evaluation of specific nutritional factors as intervention targets in randomised controlled trials. We describe possible biases when using MR, and methodological developments aimed at investigating and potentially overcoming these biases when present. Lastly, we consider the use of MR in identifying causally relevant nutritional risk factors for various cancers in different regions across the world, given notable geographical differences in some cancers. We also discuss how MR results could be translated into further research and policy. We conclude that findings from MR studies, which corroborate those from other well-conducted studies with different and orthogonal biases, are poised to substantially improve our understanding of nutritional influences on cancer. For such corroboration, there is a requirement for an interdisciplinary and collaborative approach to investigate risk factors for cancer incidence and progression.

## Introduction

Approximately 40% of cancer cases and cancer deaths in high-income countries are thought to be explained by known lifestyle, environmental, and clinical risk factors [1–3]. These risk factors include aspects of diet (e.g., alcohol consumption; eating high amounts of red and processed meat; or diets low in fruits, vegetables, wholegrains and dietary fibre) and other related lifestyle factors (e.g., overweight and obesity, physical inactivity, smoking and metabolic factors) that could account for 15–20% of total cancer cases and deaths [1–5]. Thus, an important element in the prevention of cancer and its progression is to reduce exposure to such potentially modifiable dietary and lifestyle risk factors. The World Cancer Research Fund (WCRF) now provides recommendations for cancer prevention based on comprehensive reviews of existing studies which focus on nutritional behaviour coupled with management of body weight and regular physical activity (Box 1) [2].

**Box 1:** World Cancer Research Fund (WCRF) cancer prevention recommendations

• *Be a healthy weight* - keep your weight within a healthy range (i.e., a body mass index [BMI] between 18.5 and 24.9 kg/m^2^) and avoid weight gain in adulthood
• *Be physically active* - walk more, sit less and be at least moderately physically active (i.e., increase your heart rate to about 60–75% of its maximum) every day
• *Eat wholegrains, veg, fruits and beans* - make wholegrains, vegetables, fruit and pulses a major part of your usual daily diet to consume at least 30 g of fibre with at least five portions of non-starchy vegetables and fruit per day
• *Limit “fast foods”* - particularly, limit consumption of processed foods high in fat, starches or sugars including fast foods, pre-prepared dishes, confectionary, snacks and bakery foods
• *Limit red and processed meats* - eat no more than three portions of red meat (i.e., any mammalian muscle) such as beef, pork and lamb and little processed meat per week
• *Limit sugary drinks* - do not consume sugar sweetened drinks (i.e., liquids sweetened by adding sugars such as sucrose, high fructose corn syrup and sugars naturally present in honey, syrups, fruit juices and fruit juice concentrate)
• *Limit alcoholic beverages* - for cancer prevention, it is best not to drink any alcohol
• *Do not rely on supplements* - aim to meet nutritional needs through diet alone rather than with high-dose dietary supplements (i.e., a product intended for ingestion that contains a “dietary ingredient” to supplement what is usually achievable through diet alone)
• *Breastfeed your baby* - for mothers, there is strong evidence that breastfeeding (i.e., exclusively breastfeeding for 6 months and then up to 2 years of age and beyond alongside appropriate complementary foods) helps protect against breast cancer in the mother and promotes healthy growth of the infant
• *After cancer diagnosis* - follow these recommendations, if you can, and check with your healthcare provider what is right for you. All cancer survivors (i.e., those who have been diagnosed with cancer and those who have recovered from the disease) should receive nutritional care and guidance on physical activity from trained professionals

However, the primary source of evidence supporting such recommendations about risk factors for cancer are traditional observational studies, which are vulnerable to potential biases (e.g., from confounding, reverse causality, and misreporting) when estimating effects. Such biases may, in part, explain why claims of several putative protective factors for cancer prevention have failed to be supported when tested in subsequent randomized controlled trials (RCTs) [6–8]. For example, observational studies supported a link between higher levels of beta-carotene (i.e., a red pigment found in vegetables, such as carrots, that is converted to vitamin A) and reduced cancer risk, suggesting that beta-carotene and vitamin A may prevent cancer. These findings led to the promotion of vitamin supplements and diets rich in beta-carotene as a potential strategy for cancer prevention; however, randomization of individuals to beta-carotene supplementation in trials performed subsequent to these observational findings showed no clear effect on cancer risk [6–8].

There are major geographical differences and rapid changes in cancer incidence over time suggest that many environmental risk factors for cancers exist, with a potentially large proportion still yet to be discovered. However, in the absence of RCTs, which may not always be feasible or ethical, there is a requirement for improved causal inference in the identification and verification of nutritional exposures for cancer incidence and progression.

Current challenges in nutritional cancer epidemiology include a need for (i) reliable estimates of whether previously reported nutritional exposures causally affect cancer; (ii) identification of novel causal and modifiable risk factors for cancer; (iii) better understanding of biological mechanisms underpinning the effects of nutritional exposures on cancer; (iv) investigating the impact of causal risk factors across the lifecourse; (v) understanding the nutritional effects on cancer subtypes, progression, and survival; and (vi) a more diverse and global perspective, particularly within low- to middle-income countries. In this article, we review the application of Mendelian randomization (MR) to help address these challenges, updating an earlier review by Schatzkin et al*.* in 2009 [9].

## Mendelian randomization

Originally introduced within the framework of parent–offspring studies [10], MR uses germline genetic variation, usually in the form of single nucleotide polymorphisms (SNPs), that are strongly associated with putative environmental risk factors (e.g., dietary factors, behaviour, and molecular traits) to appraise the causal relationship of these factors on disease outcomes [11–13]. Groups defined by genetic variation associated with an exposure should be largely independent of confounding factors at a population-level [14]. This is because, at conception, genetic variants across the genome are randomly inherited from parents to offspring. At a population-level, this means that germline genetic variants are less likely to be associated with many environmental (non-genetic) factors that commonly confound observational associations.

Germline genetic variants (i.e., those that can be inherited) are fixed at conception and are not modified by the onset of disease (e.g., cancer development), precluding reverse causation. Given improvements in modern genotyping technologies, measurement error in MR studies is often lower in comparison to that of the nutritional exposures. Additionally, some studies of cancer are of a case–control design and, for many nutritional factors (e.g., reported intake), retrospective reporting is subject to systematic measurement error and exposure misclassification. In contrast, this is not generally true for genetic variation (or any other biological measure). While misclassification of dietary assessments (e.g., self-reported food frequency questionnaires) may be differential in relation to other traits (e.g., BMI levels), misclassification of such dietary measures would not, generally, be expected to be differential with regards to genotypic variation. Though there could be specific, but likely rare, scenarios where differential misclassification could be expected. A consequence of non-differential misclassification of dietary assessments in an MR context is that statistical power would be reduced but MR estimates should not be biased. MR can be used to explore the longer-term effects of a particular exposure, which is particularly relevant in the context of diseases with long induction periods such as cancer.

**Fig. 1:**
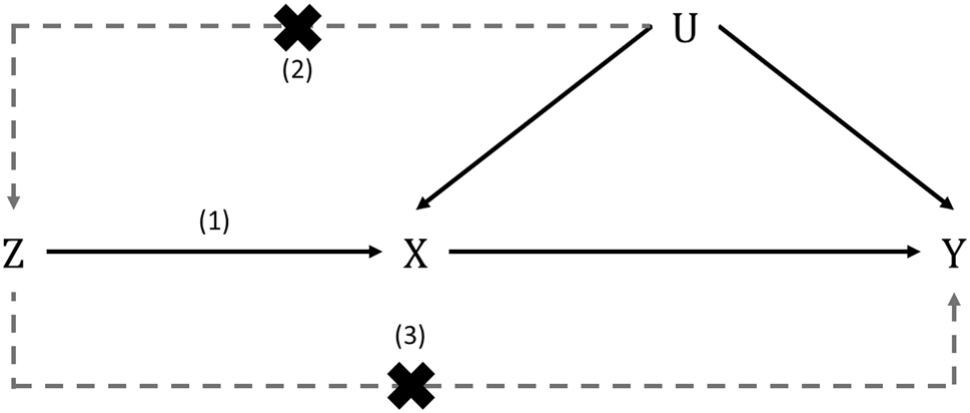
Framework and assumptions of Mendelian randomization (MR) analyses. In addition to a gene-environment equivalence assumption, MR relies on the following three core assumptions of formal instrumental variable analysis (in addition to those described as the “homogeneity”, “monotonicity” and “no effect modification” assumptions): (1) the “relevance” assumption—the genetic variant(s) being used as an instrument (Z) is robustly associated with the exposure (X); (2) the “independence” or “exchangeability” assumption—there are no common causes of the genetic variant(s) and outcome (e.g., population substructure, assortative mating and dynastic effects); and (3) the “exclusion restriction” assumption—there is no independent pathway between the genetic variant(s) and outcome (Y) other than through the exposure (X)—also known as horizontal pleiotropy

Fundamentally, MR requires a “gene-environment equivalence” assumption, namely that downstream physiological effects of modifying an exposure are the same whether they are genetically or non-genetically triggered (see Davey Smith 2012 for more details [15]) [10]. Formal applications of MR require three core IV assumptions to be met to provide a valid test of the causal null hypothesis (Fig. 1): (1) the genetic variant(s) being used as an instrument is associated with the exposure (the relevance assumption); (2) there are no common causes of both the genetic variant(s) used as an instrument and the outcome of interest (independence assumption); and (3) there is no independent pathway between the genetic variant(s) and outcome other than through the exposure (exclusion restriction assumption). In the context of the exclusion restriction assumption, the presence of a direct effect of a genetic variant used as an instrument on an outcome (i.e., not mediated through the exposure of interest) is commonly termed “horizontal pleiotropy”. In the presence of time-varying effects, however, these three assumptions are not sufficient to test all causal null hypotheses [16]. As previously shown by Swanson et al. in time-varying settings, MR estimates may only be able to provide evidence concerning the specific null hypothesis that altering an exposure at any time would have no effect on a particular outcome across all individuals.

Additional assumptions of homogeneity, monotonicity and “no effect modification” are required to quantify the average causal effect (Fig. 1). Briefly, the homogeneity assumption requires that the causal effect of an exposure on an outcome is constant across all individuals in a study, which may not be biologically plausible [17, 18]. As an alternative to estimating an average causal effect in an entire population, a local average effect can be estimated in a subgroup of that population under a weaker assumption of monotonicity (i.e., the direction of effect of a particular exposure within varying levels of the exposure-related IV is in the same direction for all individuals), although the subgroup is not defined. Another alternative to the homogeneity assumption is the “no effect modification” assumption, which requires that an instrument does not modify the effect of an exposure on an outcome differentially within levels of the exposure (e.g., through SNP-SNP or SNP-environment interactions). If the “no effect modification” assumption is met, an average causal effect can still be estimated even when an outcome is heterogenous (i.e., does not meet the “homogeneity assumption”). In addition to permitting quantification of average causal effects, the presence of effect modification in an analysis can also inform on potential biological pathways underpinning effects (discussed further in “Challenges and sources of bias in MR analyses”).

Taken together, if the above assumptions hold, any differences in an outcome by genotypic groups associated with an exposure can therefore be attributed to differences in that exposure [19]. MR has been developed and can be used in a multitude of scenarios; for example, with individual-level participant data (i.e., usually termed “one-sample MR”) and with summary-level data (i.e., usually termed “two-sample MR”), each with its own strengths and limitations. For more details on MR assumptions and methodology, please see our MR Dictionary (https://mr-dictionary.mrcieu.ac.uk/), the guidelines for performing MR analyses, and the STROBE-MR [20–22].

## Applied examples of how MR can contribute to understanding nutritional determinants of cancer

Genome-wide association studies (GWASs) have identified genetic variants that are robustly associated with a growing number of dietary factors and nutritional biomarkers (Box 2). Such GWASs have enabled the application of MR to improve understanding of nutritional exposures influencing cancer risk and progression studies in nutritional epidemiology. Three illustrated examples are described below.

**Box 2:** Examples of genetic variants associated with nutritional and nutritionrelated factors

**Genetic variants related to intake**
• *ALDH2* and *ADH1B* with alcohol intake
• *LCT* and *MCM6* with milk and dairy intake
• *CYP1A1*, *CYP1A2* and *AHR* with coffee and caffeine
• Macronutrients (e.g., the 21 loci published by the Social Sciences Genetic Association Consortium [SSGAC] [23])
** Genetic variants related to circulating biomarkers**
• Iron, ferritin, transferrin and transferrin saturation
• Alpha- and beta-carotene, retinol
• Calcium
• Blood-based metabolites (e.g., those published by Kettunen et al. [24] and Shin et al. [25] for nuclear magnetic resonance and mass spectrometry platforms, respectively)
**Genetic variants related to biologically relevant genes**
• *MTHFR* with folate
• *VDR* with vitamin D
**Genetic variants related to nutrition-related factors**
• e.g., Body mass index (e.g., the 941 published by the Genetic Investigation of ANthropometric Traits [GIANT] consortium) [26]
• The human gut microbiome (e.g., those published by Hughes et al. [27] and the MiBioGen consortium [28])

### Selenium and prostate cancer risk

Several prospective studies reported inverse associations of dietary, serum and toenail selenium with risk of prostate cancer [27–31], complemented by in vitro evidence [32, 33]. This led to the Selenium and Vitamin E Cancer Prevention Trial (SELECT) [34, 35], in which 35,533 healthy middle-aged men were randomized in a 2 × 2 factorial design to daily supplementation of selenium, vitamin E or both, with prostate cancer onset after 12 years as the primary outcome. However, the trial was terminated prematurely by the data monitoring committee after 5.5 years due to indication of an increased risk of prostate cancer in the vitamin E supplementation group along with lack of efficacy and suggestion of possible carcinogenic (i.e., increased rates of high-grade prostate cancer) and adverse metabolic effects (some evidence of increased rates of type 2 diabetes) in the selenium supplementation group [34, 35]. Though it remains unclear as to what accounted for differences between findings from observational analyses and SELECT for selenium, it is plausible that residual confounding in the former may have driven the apparent protective observational associations reported [36, 37].

Following publication of findings from SELECT, an MR analysis using 11 SNPs associated with blood selenium was undertaken to appraise the relationships of circulating selenium with overall and advanced prostate cancer risk [38]. In analyses scaled to mimic the blood selenium-raising effect of selenium supplementation versus placebo in SELECT (i.e., a difference of 114 μg/L), there was little evidence that circulating selenium was associated with prostate cancer (odds ratio [OR] 1.01; 95% CI 0.89, 1.13). Consistent with adverse effects suggested in secondary analyses of SELECT, there was weak evidence that higher circulating levels of selenium increased risk of advanced prostate cancer (OR 1.21; 95% CI 0.98, 1.49) and type 2 diabetes (OR 1.18; 95% CI 0.97, 1.43). These findings, coupled with those from SELECT, suggest that selenium supplementation is unlikely to prevent prostate cancer and that long-term selenium supplementation may increase the risk of advanced prostate cancer and type 2 diabetes. In addition, this example illustrates how the use of MR can provide additional insight into the potential efficacy of an intervention and thus inform policy when there is uncertainty remaining after a trial is stopped early. Given costs involved in developing SELECT (i.e., approximately $114 million U.S. dollars), this example also demonstrates how MR could be employed in the future as a time-efficient and inexpensive first step for prioritising or deprioritising interventions to be taken forward to testing in a RCT.

### Adiposity and site-specific cancer risk and progression

Recent MR studies have helped to refine the understanding of the role of body mass index (BMI) in the development of multiple cancers by suggesting substantially larger effect sizes for associations across multiple cancer sites, compared to those reported in the observational literature. The MR estimates for a 5 kg/m^2^ increment in BMI as proxied by 714 independent germline genetic variants was approximately two to four-fold higher than the WCRF pooled observational multivariable regression estimate for most obesity-related cancers, including cancers of the kidney (MR RR 1.59: 95% CI 1.45, 1.74 and observational RR 1.30; 95% CI 1.25, 1.35), endometrium (MR RR 2.06; 95% CI 1.89, 2.21 and observational RR 1.50; 95% CI 1.42, 1.59), pancreas (MR RR 1.47; 95% CI 1.31, 1.66 and observational RR 1.10; 95% CI 1.07, 1.14), and colorectum (MR RR 1.44; 95% CI 1.22, 1.70 and observational RR 1.05; 95% CI 1.03, 1.07) [39].

Smaller magnitudes of effect in observational analyses may reflect regression dilution bias from single time point measurements of BMI or reverse causation due to cancer-induced weight loss [40–43]. In contrast, MR estimates reflect accumulated exposure to higher mean BMI across the life-course course and are unlikely to be influenced by reverse causation. The MR results suggest therefore that the cancer burden attributable to higher BMI is likely to have been underestimated. This is important as policy makers can use this evidence to strongly promote societal-wide interventions aimed at maintaining a healthy weight.

In addition to cancer incidence, some MR analyses have examined the association of BMI with measures of cancer prognosis though such analyses can present additional methodological challenges (e.g., collider bias, discussed below in the Sect. “Developments to mitigate the challenges of MR of nutrition in cancer”). For example, an analysis of 36,210 individuals (2,475 breast cancer deaths) found evidence to corroborate some conventional observational analyses in support of an effect of BMI on breast cancer-specific survival among women with oestrogen-receptor (ER) positive breast cancer (hazard ratio (HR) per unit increase in a BMI-related genetic risk score (GRS) 1.11; 95% CI 1.01, 1.22), but not ER negative breast cancer (HR 1.00; 95% CI 0.89, 1.13) [44–46]. In MR analyses based on 46,155 participants (6,998 cancer deaths) in the UK Biobank with a cancer diagnosis, BMI was associated with an increased risk of overall cancer mortality (OR per SD increase in BMI 1.28; 95% CI 1.16, 1.41).

### Vitamin D and cancer risk

Meta-analyses of prospective observational studies have supported a role of low 25-hydroxyvitamin D levels [25(OH)D], the primary circulating form of vitamin D, in overall cancer incidence along with risk of some site-specific cancers, most notably colorectal cancer [47–49]. Whether these findings represent an effect of 25(OH)D itself, a potential common cause of lower 25(OH)D and cancer risk (e.g., cigarette smoking, excess adiposity or physical inactivity), or reverse causation is unclear and, as such, whether vitamin D supplementation is a chemoprevention agent cannot be established from these studies [50]. In contrast, MR studies found little evidence that circulating 25(OH)D affected risk of several cancers [51–54], in agreement with two large vitamin D supplementation trials [55, 56]. These findings collectively suggest that vitamin D supplementation should not be recommended as a strategy for cancer prevention.

### Insights from discordance between MR studies

In contrast to studies of cancer risk, MR analyses of vitamin D and cancer survival have been inconsistent. An early MR analysis in 95,766 Danish participants (2,839 cancer deaths) suggested that higher levels of vitamin D (instrumented using 4 SNPs in *DHCR7* or *CYP2R1*) may lower risk of cancer mortality (OR per 20 nmol/L higher plasma 25(OH)D: 0.70; 95% CI 0.50, 0.98), consistent with a meta-analysis of five trials (*N* = 1,591 deaths) showing that vitamin D supplementation (achieving a 54–135 nmol/L increase in circulating levels of circulating 25(OH)D in the intervention group) reduced total cancer mortality [relative risk (RR) 0.88; 95% CI 0.78, 0.98] [55, 57].

However, a more recent and larger MR analysis in UK Biobank (438,870 participants, 6,998 cancer deaths) found little evidence that circulating vitamin D (instrumented using 5 SNPs in *GC*, *CYP2R1*, *DHCR7*, or *CYP24A1*) influenced cancer mortality [OR per 20 nmol/L higher plasma 25(OH)D: 0.97; 95% CI 0.84, 1.11] [58]. Baseline levels of plasma 25(OH)D in the Danish analysis and in UK Biobank (as reported elsewhere) were similar, suggesting that discordance across MR studies is unlikely to reflect a relative vitamin D deficiency among Danish participants (i.e., reflecting the more northern latitude of this country) [59]. It is possible that differences in findings could reflect differences in instrument construction across studies (and, thus, differences in the likelihood of introducing horizontal pleiotropy into analyses). For example, when the authors of the UK Biobank analysis re-examined the association between 25(OH)D levels and overall cancer mortality using two of four (independent) SNPs used in the Danish analysis, point estimates generated were more similar to the Danish analysis (OR 0.88; 95% CI 0.71–1.11), though imprecisely estimated. While instruments across both analyses were constructed from biologically plausible gene regions for 25(OH)D levels (e.g., *DHCR7*, *CYP2R1*), the few SNPs available to instrument this trait meant that comprehensive sensitivity analyses (see Table 1) either could not be performed or were under-powered for these analyses. These examples therefore highlight potential challenges of reliably testing IV assumptions and, thus, obtaining reliable causal inferences when there are few SNPs in an instrument. Re-evaluating associations between 25(OH)D and cancer mortality using a recent GWAS of 417,580 individuals that identified 143 loci associated with 25(OH)D concentrations may provide the opportunity to test the robustness of findings more comprehensively to MR assumption violations and thus help to potentially reconcile conflicting findings from these analyses [60].

**Table 1:** Overview of sensitivity analyses available to examine evidence of violations of Mendelian randomization assumptions

Method	Purpose	What it does	Assumptions	Strengths	Limitations
MR-Egger regression and intercept test	Examines invalidation of the third MR assumption (i.e., horizontal pleiotropy). Specifically, this method tests for the presence of directional pleiotropy (MR-Egger intercept test) and the robustness of findings to directional pleiotropy (MR-Egger regression)	Performs a weighted generalized linear regression of the SNP-outcome effect estimates on the SNP-exposure effect estimates with an unconstrainted intercept term. If the InSIDE and NOME assumptions are met, the intercept term can provide a formal statistical test for directional pleiotropy and the slope generated from MR-Egger regression can provide an effect estimate that is adjusted for directional pleiotropy	InSIDE, NOME	Permits unbiased causal effects to be estimated even when all variants are invalid IVs	Sensitive to outliers; requires the InSIDE assumption to hold; low statistical power in the presence of no invalid instruments
Weighted median [61]	Examines invalidation and robustness of findings of the third MR assumption (i.e., horizontal pleiotropy)	Individual SNP effect estimates are ordered and weighted by the inverse of their variance. Providing at least 50% of the instruments are valid, the weighted median of this distribution is taken as an unbiased estimate of the causal effect	The median estimate (weighted by precision of SNPs) is unaffected by horizontal pleiotropy	Greater statistical power than MR-Egger; does not require the InSIDE assumption	Requires at least 50% of the information from variants to come from valid IVs
Weighted mode [62]	Examines invalidation and robustness of findings of the third MR assumption (i.e., horizontal pleiotropy)	Individual SNP effect estimates are ordered and weighted by the inverse of their variance. Providing the ZEMPA assumption is satisfied, the weighted mode generates a causal estimate using the mode of a smoothed empirical density function of the distribution of weighted SNP effect estimates	ZEMPA	Can generate unbiased causal estimates even when many SNPs in an instrument are invalid	Lower statistical power to detect causal effects than weighted median, under the condition of no invalid instruments; sensitive to bandwidth parameter
MR-CAUSE [63]	Examines invalidation and robustness of findings of the third MR assumption (i.e., horizontal pleiotropy)	Compares the expected log pointwise posterior density (i.e., estimate of how well the posterior distribution of a model is expected to predict a new set of data) under three models: a “sharing model” (i.e., permitting horizontal pleiotropy but no causal effect between traits), a “causal model” (i.e., permitting horizontal pleiotropy and assuming a causal effect), and a “null model” (i.e., neither a causal nor shared factor)	Assumes a single unobserved shared factor between two traits of interest	Can account for both correlated and non-correlated horizontal pleiotropy. Greater statistical power than MR-Egger and weighted mode when there is a true causal effect and no correlated horizontal pleiotropy	Inferior control of false positive rate in the presence of no causal effect and 0 to 50% of variants acting through a shared factor, as compared to MR-Egger and the weighted mode. Has somewhat lower statistical power than the weighted median approach when there is a true causal effect and no correlated horizontal pleiotropy
Multivariable MR [64]	Examines invalidation and robustness of findings of the third MR assumption (i.e., horizontal pleiotropy)	Performs a weighted generalized linear regression with adjustment for measured horizontal pleiotropy between instruments and outcomes	Requires that there are at least as many genetic instruments available as there are exposures	Can adjust estimates for the presence of measured horizontal pleiotropy	There can still be horizontal pleiotropy through variants having effects on unmeasured outcomes that are independent to the exposure of interest
Outlier detection tests (e.g., MR-PRESSO [65], Radial MR [66])	Remove or down-weight genetic variants that are outliers in an MR analysis	Differing methods	Perform better when a large proportion of variants are not horizontally pleiotropic	Explicitly remove or down-weight contributions of outliers that may be indicative of IV assumption violations; can improve statistical efficiency of models	Residual directional pleiotropy can remain after removing or down-weighting outlying SNPs; methods are underpowered when few SNPs are available; interpretation of an “outlying” variant may be ambiguous when there are few SNPs available in a multi-SNP instrument
Colocalization	Examines whether an association of a SNP with two or more traits represents both traits sharing a single causal variant or distinct causal variants in linkage disequilibrium	Differing methods	Some methods assume at most a single causal variant within the region for two traits examined	Can rule out findings being driven by two traits having distinct causal variants in high linkage disequilibrium	Can be underpowered for disease outcomes as compared to molecular traits
Steiger filtering/reverse direction MR [67]	Examines whether the association of a variant with two traits (e.g., A and B) represents a proximal effect of the variant on trait A which then influences trait B or vice versa. Reverse direction MR attempts to understand direction of effect between two traits	Steiger filtering compares the proportion of the variance explained in the exposure and outcome by SNPs used as instruments to help establish directionality between associations	No horizontal pleiotropy	Can help to elucidate the direction of association between two traits	Steiger filtering is sensitive to differences in measurement error and sample size across traits examined

*InSIDE* INstrument Strength Independent of Direct Effect, *NOME* NO Measurement Error, *ZEMPA* ZEro Modal Pleiotropy Assumption

## Challenges and sources of bias in MR analyses

There are several important methodological challenges to and unique sources of bias that must be considered when applying MR to evaluating the causal role of nutritional factors in cancer.

Firstly, such analyses are limited to nutritional traits that have been shown to robustly associate with germline genetic variants. Although the number of GWASs of nutritional factors has increased in recent years, the sample sizes of these studies are often relatively limited, and thus for many factors there are relatively few established genetic variants that can be used as instruments in an MR framework. Large-scale collaboration across GWASs with measures of nutritional traits (as has been achieved, for example, for “energy balance” as indicated by adiposity within the GIANT consortium [68]) will facilitate the continued discovery of genetic variants that influence nutritional traits.

Secondly, MR studies of nutritional factors often have insufficient statistical power to detect modest effect sizes, because of the moderate number and size of effects of genetic variants for individual nutrients and small heritable components of many nutritional exposures [69]. Power for MR analyses has generally increased with larger GWASs of nutritional exposures and site-specific cancers and the application of two-sample MR which leverages independent genotyped samples with dietary and cancer data to estimate causal effects, even in the absence of complete phenotypic data across both samples [70]. However, one potential trade-off from constructing instruments using increasingly larger GWASs is the possibility that these SNPs are more likely to be horizontally pleiotropic [71].

Thirdly, as is the case with many non-nutritional exposures, limited biological understanding of the mechanisms underlying the associations of genetic IVs with nutritional exposures can complicate or undermine interpretation of findings. For example, in an early MR analysis of alcohol intake and oesophageal cancer, understanding the dual role of an *ALDH2* genetic variant (used to instrument alcohol intake), which influences both alcohol intake and acetaldehyde metabolism, was essential in ensuring correct interpretation [72].

Specifically, the *ALDH2* locus encodes an enzyme (aldehyde dehydrogenase) that metabolizes acetaldehyde, the principal metabolite of alcohol and a carcinogen [73]. Each copy of the *ALDH2* *2 allele produces an inactive protein subunit that is unable to metabolise acetaldehyde, resulting in markedly higher acetaldehyde levels in *1*2 heterozygotes and *2*2 homozygotes, compared to *1*1 homozygotes, when alcohol is consumed. Carriers of the *2 allele also experience facial flushing along with nausea and other unpleasant symptoms after consuming alcohol, and thus have reduced tolerance (and consumption) of alcohol, which is particularly severe in *2*2 homozygotes.

In a meta-analysis of seven studies of 905 oesophageal cancer cases in East Asians, individuals with the *ALDH2* *2*2 genotype were found to have a lower risk of oesophageal cancer as compared to those with a *1*1 genotype (OR 0.36; 95% CI 0.16, 0.80). This suggests that lower levels of alcohol consumption protect against oesophageal cancer risk. When individuals with a *1*2 genotype were compared to *1*1 homozygotes, the former were shown to have an elevated risk of oesophageal cancer (OR 3.19; 95% CI 1.86, 5.47). A naïve interpretation would be that individuals with moderate vs. higher alcohol consumption had an elevated risk of oesophageal cancer.

However, stratification of these results by self-reported alcohol intake revealed that there was no strong evidence for an increased risk of cancer in *1*2 heterozygotes relative to *1*1 homozygotes who abstained from alcohol (OR 1.31; 95% CI 0.70, 2.47); whereas, among self-reported heavy drinkers, there was an approximate seven-fold increase in risk (OR 7.07; 95% CI 3.67, 13.60). Therefore, the observation of an increased risk of oesophageal cancer among individuals with a *ALDH2* *1*2 genotype compared to *1*1 homozygotes suggested that the substantially elevated acetaldehyde levels in these heterozygotes likely mediated the effect of alcohol intake on oesophageal cancer.

Fourth, genetic instruments may be associated with different dimensions of the same nutritional factor or behaviour, a phenomenon termed “trait heterogeneity,” making interpretation of some MR findings challenging [11]. For example, various genetic variants related to coffee intake are also linked to caffeine metabolism [74]. In the absence of strong biological knowledge into trait complexity, this lack of specificity can make it difficult to disentangle whether observed effects are primarily driven through coffee intake (independent to caffeine metabolism) or caffeine levels or, alternatively, whether null effects observed could be driven through divergent effects of coffee and caffeine on a particular outcome.

Finally, robust conclusions from MR require that the three core IV assumptions required to test the causal null hypothesis hold true. However, only the relevance assumption is readily verifiable (Fig. 1). Instrument strength and the presence of weak instrument bias, when an instrument explains only a small proportion of variance in an exposure, is typically assessed by calculating an F-statistic, with a threshold of ≥ 10 a conventional “rule of thumb” indicating minimal weak instrument bias [75]. The presence of weak instrument bias can have differing effects depending on the analysis performed: in one-sample MR with individual-level data, weak instruments are expected to bias estimates towards the confounded multivariable regression estimate; in two-sample MR with summary-level data, weak instruments tend to bias estimates toward the null (in the absence of sample overlap).

Threats to the exchangeability assumption include confounding due to differences in population substructures, assortative mating, and dynastic effects [76]. Confounding through population substructure is typically addressed through restricting analyses to ancestrally homogeneous groups and adjusting for principal components of ancestry or using linear mixed models but [77], as GWASs increase in size, these methods may fail to capture residual confounding through increasingly subtle population substructures. Assortative mating, where there is non-random matching between spouses, can produce spurious associations and/or biased effect size estimates. Dynastic effects represent indirect effects of parental genotype on offspring genotype mediated via parental traits. For example, it is plausible that parental genetic variants could influence diet in childhood even if these are not inherited by the child (e.g., through parental feeding behaviours or the shared environment) which could have long-term effects on dietary preferences in adulthood, in turn influencing subsequent cancer risk.

Evidence is emerging that MR analyses performed in samples of unrelated individuals may be biased due to the aforementioned exchangeability violations, though this bias appears to be more pronounced for socioeconomic and behavioural traits (e.g., educational attainment and smoking status) as compared to physiological measures (e.g., circulating biomarkers) [78]. Consequently, these biases may have more relevance to MR analyses (performed in unrelated individuals) of self-reported dietary intake and dietary patterns as compared to circulating measures of nutritional factors. Within-family MR, which uses parent–offspring trios or sibship designs, is increasingly feasible and can help to correct for biases due to each of these effects [76, 79]. Indeed, the MR approach was originally introduced within the framework of parent–offspring studies [10]. Limited statistical power of studies using parent–offspring designs at the time that this approach was conceptualised (in 2003) meant that the only viable analyses that could be performed were often those that used population data in unrelated individuals which relied on the premise that the random inheritance of genetic variants from parents to offspring is reflected at population-level in these individuals [10, 80]. Sensitivity analyses to assess violations of the exclusion restriction criterion include:

- Tests for horizontal pleiotropy that is not “balanced” across an instrument (“directional pleiotropy”), such as the MR-Egger intercept term [81].
- Analyses to correct for directional pleiotropy in a regression model, such as MR-Egger regression [81].
- Analyses that can provide unbiased causal estimates in the presence of invalid instruments such as weighted median- and mode-based estimators [61, 62, 65, 66, 81–84]. These methods relax certain IV assumptions while introducing additional assumptions.

A non-exhaustive list of these sensitivity analyses along with their key aims, assumptions, strengths, and limitations is presented in Table 1 (more definitions and details in the MR Dictionary). Given differences in the assumptions required of these methods, it is often beneficial to test the consistency of findings across various sensitivity analyses.

## Developments to mitigate the challenges of MR of nutrition in cancer

Several methods that have been developed to mitigate the challenges in the design of MR studies of nutrition and cancer are highlighted below. Specifically, we discuss:

- Instrument identification
- Tissue-specificity
- Hypothesis-free investigations of the phenome
- Two-step MR for mediation analyses
- Multivariable MR for correlated traits, mediation and time-varying exposures
- Evaluating risk factors for cancer progression and collider bias in case-only studies
- Factorial MR for the combined impact of multiple nutritional exposures
- MR to examine non-linear associations.

### Instrument identification

Beyond established instruments for alcohol and milk intake (Box 2), there are currently few reliable instruments for dietary intake. Performing GWASs for broad dietary factors and behaviours has the potential threat of uncovering non-informative SNPs or SNPs that are indirectly related to diet through other sociodemographic and behavioural factors, re-introducing confounding into MR analyses. For example, a recent GWAS of dietary habits in the UK Biobank identified a variant (rs1421085) in *FTO*, an established adiposity locus, associated with a principal component-derived dietary pattern profile at genome-wide significance [69]. Naïve use of this SNP to proxy a particular dietary pattern in the context of an MR analysis examining the effect of this dietary pattern on subsequent adiposity or adiposity-related traits would be erroneous as this variant is likely to influence dietary patterns via its effect on adiposity (i.e., rather than influencing adiposity via an effect on dietary patterns). Thus, these complex relationships between genetic variation and the primary phenotype of interest need to be understood at a more basic level within the context of MR.

In contrast, instruments for circulating biomarkers relevant to dietary intake are more abundant, including, for example, variants linked to circulating levels of iron, ferritin, vitamins (e.g., alpha- and beta-carotene, retinol and vitamin C), selenium and calcium.

However, as is the case for many molecular markers, interpretation of findings when using instruments based on nutritional biomarkers may introduce their own challenges when the biology underpinning associations with genetic variants is unclear. For example, circulating biomarkers may not accurately represent the cellular concentration of that marker, such as intracellular biomarkers involved in paracrine or autocrine signalling [85]. Indeed, higher circulating levels of a particular biomarker may represent lower levels of cell uptake or cell signalling regulation in the tissue of interest, as is the case for variants in *IL6R* associated with higher circulating concentrations of interleukin-6 because of lower cellular binding of this protein to its receptor [11]. To overcome this limitation, it is possible to generate instruments from variants within well-characterised gene regions. For example, variants in *MTHFR*, which encodes the rate-limiting enzyme in one-carbon metabolism, could be used to proxy folate; similarly, variants in *VDR,* which encodes the vitamin D_3_ receptor, could be used to proxy vitamin D-related pathways. However, if variants have not been identified in GWASs, it may be difficult to generate a causal effect estimate of the exposure-cancer relationship due to unreliable quantification of the SNP-exposure association. Furthermore, publication bias in candidate gene studies calls into question the reliability of variants identified in these type of studies [86]. By extension, as in conventional molecular epidemiological analyses, circulating biomarkers may also not reflect changing consumption of related nutritional exposures. As several micronutrients (e.g., calcium) are tightly regulated, dietary intake will be unlikely to lead to detectable changes in circulating concentrations; thus, MR estimates should not be interpreted as being relative to dietary intake per se and, in general, this limits power to detect genetic contributions to these micronutrients.

GWASs of increasingly refined nutritional biomarkers and traits (e.g., using assays of xenobiotics or urinary metabolite data as biomarkers of dietary intake) will help to increase the number of genetic variants available to develop instruments for MR analyses. For example, the growing understanding of germline genetic contributors to the human gut microbiome could help to examine the potential role of microbiota in site-specific cancers [87–89]. Integration of high throughput profiling of the metabolome, proteome and transcriptome within GWASs will also permit MR studies to examine potential mediating roles of these molecular markers in linking nutritional exposures to cancer risk and progression [90–92]. Targeting these factors and pathways may be more preferable and achievable when dietary modification is challenging.

### Tissue specificity

MR analyses have historically examined associations of circulating levels of nutritional biomarkers on cancer. However, in contexts where a gene’s function is restricted to (or relevant only in) a particular tissue, MR estimates using biomarker levels measured in whole blood may not be a suitable proxy for tissue-specific expression levels or activity of a particular gene product [93, 94]. Using genetic variants associated with tissue-level gene expression (expression quantitative trait loci, eQTLs) as instruments can permit exploration of the possible tissue-specific effects of molecular traits downstream of nutritional factors on cancer outcomes. Instruments for such analyses are becoming increasingly available from the genotype-tissue expression (GTEx) consortium, which, at the time of writing, has integrated genotyping with gene expression data from 49 tissue types from 838 individuals [90, 95]. Richardson et al*.* used cis-acting eQTLs obtained from GTEx to examine associations of genetically instrumented, tissue-specific expression of 32,116 transcripts with 395 complex traits, including a range of dietary intakes and nutritional biomarkers [96]. In the context of nutritional epidemiological studies of cancer, such tissue-specific MR analysis could be used, for example, to examine whether alterations of gene expression in the colonic epithelium in relation to *n*−3 polyunsaturated fatty acid (PUFA) intake, as reported in animal models of colorectal cancer, may mediate an effect of *n*−3 PUFAs on subsequent cancer risk [96, 97]. An important consideration when performing tissue-specific MR analyses is that limited and variable sample sizes currently available for different tissues means that few reliable instruments may be available in some tissues, so that differences in findings across tissues may simply reflect differences in statistical power.

### Hypothesis-free investigations of the phenome

The integration of MR within studies that scan a comprehensive range of all measured phenotypes (otherwise known as phenome-wide association studies, MR-PheWASs) can be used to appraise causality in multiple relationships simultaneously in an agnostic manner, and so generate novel hypotheses [98–100]. MR-PheWASs can be used to characterize the potential causal downstream effects of a particular exposure and prioritise modifiable risk factors for an outcome of interest. For example, Langdon et al*.* used MR-PheWAS in a hypothesis-free scan of 486 lifestyle, behavioural, and molecular risk factors using summary statistics from 28 GWASs. The analysis corroborated previously reported effects of adiposity-related risk factors on pancreatic cancer, and identified novel risk factors, such as altered circulating levels of ADpSGEGDFXAEGGGVR*, a fibrinogen-cleavage peptide, and O-sulfo-l-tyrosine [101].

Given the increasing statistical power afforded by larger sample sizes of individual and curated GWAS databases (e.g., the GWAS Catalog [102], GWAS ATLAS [103], the IEU OpenGWAS project [104, 105] and PhenoScanner [106]) coupled with efficient analytical platforms for performing such analyses (e.g., PHESANT for UK Biobank data [107] and MR-Base [104]), MR-PheWAS are becoming more commonplace. However, MR-PheWASs require careful consideration of a multiple testing correction, which can limit power and the number of causal relationships that are potentially prioritized for follow-up. That being said, with sufficiently strong instruments, such an approach could readily be applied to investigation of the contribution of dietary intake and different cancers and other diseases [108].

### Two-step MR for mediation analyses

Two-step MR enables the estimation of mediating effects (mechanisms) linking upstream exposures with cancer risk and progression. In the first step, genetic variant(s) associated with the exposure are used in MR analyses testing the relationship between the exposure and proposed intermediate trait(s). In the second step, a second set of genetic variants, independent of those used in the first step, are used in MR analyses testing the relationship between the intermediate trait(s) and the outcome of interest. Evidence for a causal effect in both steps provides some evidence for causal mediation in the exposure-outcome relationship via this intermediary, with appropriate consideration of the assumptions of mediation and MR analyses. Two-step MR can be used to test for evidence of mediation in a known or hypothesized pathway or can be expanded (and combined with MR-PheWAS, for example) to identify and estimate the effects of potential mediators in an exposure-outcome relationship of interest. For example, MR has provided robust evidence that cigarette smoking causes alterations to DNA methylation but little evidence that DNA methylation at several CpG sites in peripheral blood play a causal role in lung cancer development, consistent with methylation at these particular sites not be relevant for lung cancer [109].

Two-step MR can be used to estimate the proportion of the total exposure-outcome effect influenced by this intermediate trait and, by combining with traditional mediation methodology or multivariable MR (MVMR; see below), both the indirect effect (i.e., the effect of the exposure on outcome only via the intermediate) and direct effect (i.e., the effect of the exposure on outcome independent of the intermediate) [110].

The method has, however, been used relatively infrequently due to requirement of large sample sizes, either individual- or non-overlapping summary-level data for all three components (i.e., exposure, intermediate and outcome traits), multiple valid instruments for exposure and valid instruments for mediator that are independent of exposure, and appropriate correction against false positives due to multiple testing. However, steps one and two have been performed separately in a range of different studies to make inferences of likely molecular mediation, without undertaking a full mediation analysis [111–113].

### Multivariable MR for correlated traits and mediation

**Fig. 2:**
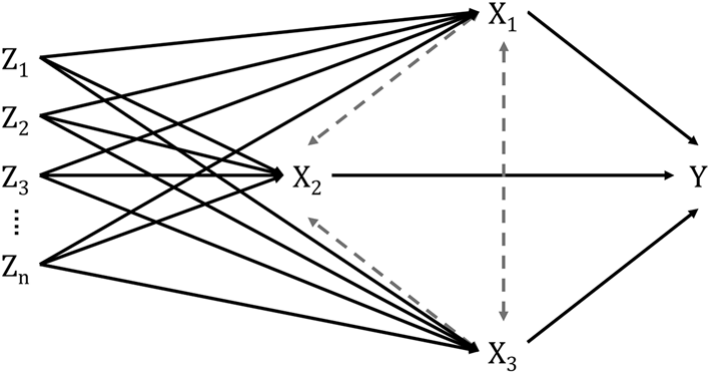
Multivariable MR for correlated nutritional factors. Multivariable MR uses multiple genetic instruments (Z_1_, …, Z_n_) associated with multiple, potentially correlated exposures (e.g., X_1_, X_2_, and X_3_) to jointly estimate the independent causal effect of each of the exposures on a particular outcome (Y). It can also be used to explore mediation following two-step MR analyses to provide a better understanding of the direct, indirect and total effects of each exposure [64, 115]

MVMR can be used to address known pleiotropy in genetic variants associated with multiple, highly correlated exposures such as lipids, metabolites, adiposity measures and macronutrients. MVMR uses genetic variants associated with multiple exposures to jointly estimate the independent causal effect of each of those correlated exposures on the outcome—the direct effect of each exposure (Fig. 2). For example, in a MVMR analysis of 6,034 oral/oropharyngeal cancer cases and 6,585 controls, Gormley et al*.* demonstrated that smoking and alcohol consumption both conferred direct effects on oral/oropharyngeal cancer risk when mutually adjusted for each other (per SD increase in lifetime smoking: MVMR OR 2.6; 95% CI 1.7, 3.9; per SD increase in drinks consumed per week: MVMR OR 2.1; 95% CI 1.1, 3.8) [114].

Used in conjunction with two-step MR, MVMR can be used in mediation analyses to estimate the direct, indirect, and total effects of interrelated traits [116, 117]. When using MVMR for formal mediation analyses, the instruments for the exposure and mediator need to be independent.

However, MVMR analyses require instruments that explain sufficient variation in each exposure conditional on the other exposures (i.e., this should be re-estimated in light of other exposures being modelled) [118]. Though, if the exposures of interest are very highly correlated, then the MVMR analysis may have very low power [119].

With the growth of studies and GWASs focusing on multiple characterisations of nutrition (e.g., intake, preferences and composition) and the inherent correlation between nutritional factors, MVMR (alongside factorial MR—see below) will be increasingly useful in the examination of the impact of nutrition on cancer aetiology and prognosis.

### Multivariable MR for time-varying exposures

Some nutritional factors may operate during a critical period of the life-course to influence cancer risk, such as the hypothesized protective role of phytoestrogen exposure during puberty on subsequent breast cancer risk [120]. Under the assumption that the relationship between a genetic variant and an exposure is constant over time (i.e., from conception to measurement of an outcome at a particular time), Labrecque and Swanson proposed that MR estimates generated using univariable models can be considered as an average “lifetime” change in this outcome measured at this time in relation to a unit change in the exposure [121]. Under this definition, if effects are time-varying, however, an MR estimate cannot reflect a “lifetime” effect, as it cannot be summarised by measuring it at any one point in time and it is advised that MVMR be employed to examine potential time-varying exposures. More recently, Morris et al. have proposed that a “lifetime” causal effect can be estimated in MR analyses using one measure of a time-varying exposure if conceptualised as the “causal effect of changing the liability [to the exposure] such that the exposure would be one unit higher at a given time” [122]. In this framework, the estimated “lifetime effect” would differ in magnitude if measured at a different point in time, but MR estimates would nonetheless be consistent with the underlying trajectory of an exposure induced by a SNP. In the presence of multiple SNPs with different time-varying effects on the exposure (i.e., leading to different estimated effect of the exposure on an outcome), MVMR could be used to disentangle the effects of different liabilities to a particular exposure on an outcome. Where MVMR is not possible to perform (e.g., where only one SNP is available to instrument an exposure or where data are not available to examine whether multiple SNPs in an instrument have differing time-varying associations with the exposure), authors should, at minimum, comment on the precise estimand of interest in an analysis and all assumptions that must be met to reliably estimate this.

By extension, in the presence of a time-varying exposure, a univariable MR analysis of an exposure at an earlier stage in the life-course (e.g., in childhood) on an outcome can generate a total effect of that exposure on outcome, which could include any effect that is mediated through that exposure at a later time point (e.g., in adulthood). In contrast to a univariable MR analysis, when genetic variants have differing effects on the exposure at different time points, MVMR can be used to estimate direct effects of time-varying exposures on disease outcomes, decomposing effects that are operating at more than one time point. Richardson et al*.* recently used this approach to disentangle the effects of childhood and adulthood body size on breast cancer risk [123]. The authors reported strong evidence for a protective direct effect (not via adult body size) of larger childhood body size on breast cancer risk (OR 0.59; 95% CI 0.50, 0.71) with less evidence for a direct effect of adult body size (OR 1.08; 95% CI 0.93, 1.27). An important caveat of MVMR analysis applied to time-varying exposures is that large sample sizes for measures across different time points are required.

### Evaluating risk factors for cancer progression and the potential for collider bias

MR studies of cancer progression could inform trials testing adjuvant therapies in a cancer survival setting [124]. In recent years, there has been a growth in GWASs examining germline genetic contributions to measures of disease prognosis and survival, as opposed to incidence. This is of particular importance in studies of cancer, as there is evidence suggesting that the effects of particular exposures may differ between incidence of cancer and subsequent progression once cancer has occurred [104]. Whilst GWASs of cancer prognosis have generally uncovered few genome-wide significant loci to date, explained in part by the relatively smaller sample sizes of these studies compared to those for disease incidence, these summary-level data can still be valuable in MR for identifying causal risk factors for cancer progression. As such, MR has been used to examine the causal roles of alcohol consumption on prostate cancer mortality in men with low-grade disease, coffee consumption on prostate cancer progression and BMI on breast cancer survival [44].

**Fig. 3:**
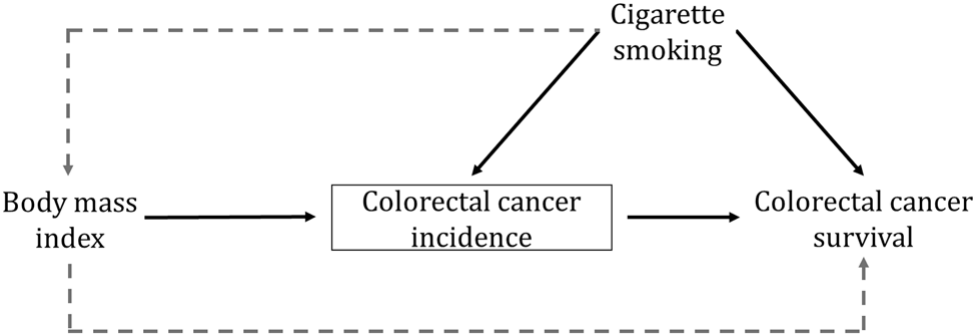
Directed acyclic graph illustrating selection bias in a Mendelian randomization analysis of cancer prognosis. In this example, estimating the causal effect of body mass index on colorectal cancer survival, the sample is restricted to colorectal cancer cases. Conditioning analyses on colorectal cancer incidence (i.e., case status, a collider in this scenario) could generate a spurious association between two causes of colorectal cancer incidence (i.e., body mass index and cigarette smoking). This then induces an association between body mass index and colorectal cancer survival (via cigarette smoking) even in the absence of a true causal relationship between these two traits in the target population

However, restricting prognostic study samples to individuals with cancer can cause “collider bias”, which can potentially induce an attenuation, reversal or overestimation of associations between otherwise independent exposures for cancer progression when adjusting or selecting for a common consequence of those two exposures [124–126]. When restricting the study sample to those who have cancer in prognostic studies, all independent risk factors for cancer incidence become associated with each other. If one or more of these risk factors is also a prognostic factor for cancer, this can potentially lead to a spurious associations between exposure-associated genetic variants and cancer prognosis. Collider bias is an issue for both conventional observational and MR approaches, and, to address it, any common causes of cancer incidence and progression need to be measured and controlled for [124, 127] (see Fig. 3, [128]).

The development of methods for mitigating collider bias in case-only progression studies is an active area of research, including the use of directed acyclic graphs (DAGs) of correlates of both cancer incidence and progression and notable methodological developments that adjust for collider bias in an MR setting [124, 129]. Firstly, if associations exist between exposure-related genetic variants and common causes of cancer incidence and progression, adjusting for these common causes may mitigate collider bias [124]. This not only requires individual-level data but is itself subject to unmeasured confounding and measurement error. Secondly, quantifying and correcting the induced bias is possible using analytical formulae proposed by Yaghootkar et al. or inverse probability weightings. However, these methods require certain population-level parameters, such as cancer prevalence and the effects of genetic variation and confounders on cancer incidence, to be quantified. Thirdly, Dudbridge et al. recently proposed a method that uses the residuals from the regression of genetic effects of cancer prognosis on the genetic effects of cancer incidence to eliminate (when the genetic effects on disease incidence and progression are independent) or, more realistically, reduce this bias [126, 130, 131].

Another potential source of collider bias is the presence of survival bias (i.e., selective survival of participants prior to study enrolment) [132]. This may be particularly problematic for cancer GWASs where participants tend to be middle-aged or elderly. In a hypothetical MR study examining the effect of BMI on cancer risk, if genetic variants used to proxy BMI also influence mortality prior to study enrolment, conditioning on surviving to study entry can induce an association between these genetic variants and other common causes of survival into a study and cancer risk. Additionally, selection into studies (particularly large cohort studies or biobanks with low response rates) is likely to have a considerable impact on the representation of the study (e.g., only 5% of those invited to UK Biobank were enrolled and are therefore likely not an accurate representation of the wider UK). If genetic variants used to instrument an exposure in an MR analysis also influence participation in a study, this could also introduce collider bias, as conditioning on study participation can induce an association between these genetic variants and other common causes of participation in the study and an outcome of interest.

### Factorial MR for the combined impact of multiple nutritional exposures

Factorial MR methods estimate the combined causal effects of two or more exposures on disease outcomes [10, 133–136]. Similar to factorial RCTs, factorial MR provides estimates of risk of disease outcome in the presence of two or more causal factors that act independently of each other (i.e., no statistical interaction) and the ability to explore potential synergistic or antagonistic interactions (i.e., where the effect of two risk factors is different to what would be expected based on independence and risk factor prevalence). Previous examples have evaluated the combined and individual effects of genetic polymorphisms at two loci encoding drug targets in the *NPC1L1* and *HMGCR* loci (that lower low-density lipoprotein cholesterol (LDL-c)) with CHD risk [135], and tested the individual and combined effects of BMI and alcohol consumption on liver disease [133].

Challenges include finding studies with adequate statistical power due to the requirement of large-scale, (usually) individual-level data, and justifying the dichotomisation or categorisation of multiple risk factors for translation to realistic scenarios. Whilst factorial MR can identify whether two independent exposures might interact and have a combined effect of public health importance, extensions using MVMR can provide a more efficient approaches for estimation of statistical interaction [137]. Furthermore, if the group upon which stratification is based is a common consequence of exposure-related genetic variants and common causes of the exposure and outcome of interest, stratification for factorial MR analyses may induce spurious associations (in either direction) via collider bias.

### MR to examine non-linear associations

Almost all MR studies to date have estimated linear effects assuming that increasing the exposure, at any level, leads to the same increase in outcome. However, observational data may suggest a non-linear relationship such as a J- or U-shaped association. For example, an inverted U-shaped association was reported between circulating 25(OH)D and skin cancer risk, suggesting that intermediate levels of 25(OH)D confer greater risk than either low or high levels [138]. Here, a linear analysis may suggest little overall effect. A naïve approach in such cases would be to stratify individuals by exposure level and perform MR within each stratum, but this could create collider bias.

Instead, methods using individual-level data have been proposed to stratify individuals on the “instrument-free exposure”, which can be used to examine the potential non-linearity of effects of nutritional risk factors on cancer [139, 140]. Such methods have recently become possible with BMI in relation to mortality risk in the UK Biobank and Norwegian HUNT studies [137, 141–143]. These approaches are promising but may be under-powered, and some technical challenges remain [144]

## International perspectives

Many existing GWASs combine multiple independent studies into large consortia, involving substantial international collaboration (e.g., PRACTICAL, GECCO and ILCCO-TRICL for cancer [145–147]). Despite this, a substantial majority of GWAS analyses focus on individuals of European descent or adjust for heterogeneity arising from ancestral diversity or population structure [148]. Therefore, MR analyses have predominantly focused on populations of European ancestry. Given heterogeneity between samples of varying ethnic and ancestral diversity (with regards to differential genetic architectures, cancer prevalence and exposure levels [145–147]), challenges associated with this approach include questions over the generalizability of genetic variants found in predominantly European GWASs and their use within MR analyses in other non-European populations. For example, comparing GWASs of alcohol intake in the UK Biobank and Kadoorie Biobank, obtained genetic variant effect sizes (either individually or in combination) differ substantially both between studies and even between sexes and geographic regions within east Asian populations, likely due to cultural differences in alcohol intake between and within these diverse populations [149, 150]. Therefore, there is a growing need for large-scale GWASs in populations of predominantly non-European ancestry and non-Westernized contexts to increase our ability to detect SNPs for the appropriate application of MR within different ancestrally and culturally diverse populations.

Within the last 5 years, there has been greater use of the ethnic diversity within existing GWASs and consortia and an increase in the number of cancer GWASs in non-Europeans, which have then been included in trans-ethnic meta-analyses [145–147, 151–157]. For example, Lu et al. conducted a GWAS of colorectal cancer specifically within East Asians from 14 studies in the Asia Colorectal Cancer Consortium (*n* = 2,775 cases and *n* = 47,731 controls) [153]. Schmit et al*.* conducted a GWAS of colorectal cancer in individuals of European descent (*n* = 49,900 cases and *n* = 79,247 controls) and examined the generalizability of discovered variants in East Asians, African Americans and Hispanics (*n* = 12,085 cases and *n* = 22,083 controls) [151]. As the number of trans-ethnic GWASs increase, so will the ability to compare results derived from MR analyses across contexts and validate results across different ethnicities and ancestries.

Access to data from international, non-European cohorts enables the application of MR to appraise risk factors that may be specific to non-Westernized cultures. For example, evidence from observational studies conducted in Northern and Eastern India suggests that adulterated mustard oil (e.g., those containing high levels of sanguinarine or diethyl nitrosamine, known carcinogenic adulterants [158]) increases the risk of gallbladder cancer, potentially driven by its pro-inflammatory properties [159, 160]. With information on the genetic contribution to the behaviour of consuming mustard oil, the circulating metabolic response to eating mustard oil or even the component parts of mustard oil (i.e., levels of sanguinarine or diethyl nitrosamine), MR analyses could test whether consuming mustard oil or exposure to its components has a causal impact on gallbladder cancer in Indian populations. Similarly, knowing more about the genetic contribution to preference and consumption of spicy foods may enable further causal analyses of the observational relationship between consumption of spicy foods and mortality found across geographically diverse areas of China [161].

With the recent development of large international biobank studies in non-European settings (e.g., China Kadoorie Biobank, Biobank Japan, GeNuIne Collaboration and the NIHR-funded South Asia Biobank [162–165]) and in low-to-middle-income settings, the opportunity to expand GWASs and MR analyses to obtain further insights into the role of nutrition in cancer from an international context will improve. Similarly, the ability to triangulate MR findings from these contexts with complementary observational studies and RCTs will improve causal inference.

## Translational considerations

When considering translating findings from MR analyses to potential population interventions, there are several important issues to consider. Firstly, since MR estimates of some nutritional factors may represent the effects of longer-term effects to a particular exposure, effect estimates obtained in such analyses may be of a larger magnitude than those obtained in a clinical trial of a relatively shorter intervention or follow-up period. To refine understanding of necessary intervention lengths and/or follow-up periods required for a nutritional intervention to confer an effect on cancer risk in a hypothetical trial, conventional multivariable regression analysis could be used to examine the association of a nutritional exposure, with indication of a potential causal relationship from MR, with cancer risk over variable lengths of follow-up. The identification of molecular biomarkers which may mediate the effect of a hypothetical intervention on cancer risk (e.g., as identified through MR analysis) could also be used to establish short-term intermediate endpoints in the setting of a feasibility trial which in turn could guide investigators in planning adequate intervention and follow-up periods of a subsequent trial examining cancer risk as the primary endpoint.

Secondly, univariable MR may not inform on a critical or sensitive period of the life-course over which an exposure is operating. Where such life-course-specific effects are suspected, MVMR could (in principle) be used to identify these periods and, thus, potentially inform on the most appropriate timing for testing an intervention in a randomized trial. However, such an analysis would require the presence of genetic variants which confer effects over differing parts of the life-course, which may not be the case for most nutrition-related exposures. Thirdly, as in conventional observational analyses, effect estimates obtained in MR analyses may not be relevant to the population to be targeted in an RCT. For example, MR analyses performed in vitamin D replete populations may not be of relevance to vitamin D deficient populations. Access to individual-level genetic association data can facilitate exploration of causal hypotheses that are targeted to particular populations of interest.

A two-stage randomisation analysis design has been proposed in which effects of interventions on long-term clinical outcomes are predicted via changes in intermediate biomarkers examined in feasibility trials (i.e., small-scale, preliminary trials which aim to assess the acceptability and viability of interventions). Here, differences in intermediate trait levels across intervention and control arms of a feasibility or early-stage RCT (first stage) are genetically instrumented and then tested for association with a disease outcome of interest using MR (second stage) [166]. Such an approach permits extension of findings from feasibility trials, which are often unable to establish effects of interventions on clinical endpoints due to their limited duration, to potential downstream effects on cancer risk or progression. Beynon et al*.* used findings from a 6-month feasibility trial, which reported an effect of dietary lycopene interventions on levels of the metabolites acetate, pyruvate, valine and docosahexaenoic acid in 133 men with raised prostate-specific antigen (PSA) levels who did not have prostate cancer [167]. Genetic instruments to proxy these metabolites were then constructed and tested for their association with prostate cancer risk using genetic data on 44,825 cases and 27,904 controls in the PRACTICAL consortium. Each SD increase in genetically instrumented pyruvate was associated with a 29% (95% CI 3, 62%) higher odds of prostate cancer, suggesting one potential pathway through which nutritional lycopene interventions could influence prostate cancer risk. An important consideration in two-stage randomisation analyses is that samples included in both stages of such analyses are representative of the same underlying population.

## Conclusions

MR is now a well-established method in the epidemiologist’s toolbox for interrogating causal relationships. The rapidly increasing wealth of genotype data on well-characterised populations continues to enhance the potential for well-powered MR studies. Advances in the way diet and its nutritional components are measured can be exploited to good effect using this method. The measurement of molecular phenotypes that can proxy some nutritional exposures (i.e., proteins, lipids, amino acids, etc.) is more widespread. These ’omic measures can be readily coupled to genotype data and thus expand opportunities for MR and identification of new intervention targets aimed at molecular intermediates. Improvements in MR methods, including an increasing panel of sensitivity analyses which interrogate and overcome certain biases, provide a more robust basis to advance causal claims. There now exist semi-automated approaches to analyses (and readily accessible platforms) that can help to search thousands of potential nutritional cancer relationships and prioritise areas of most interest. MR can also be applied, in certain instances, to predict the possible outcome of hypothetical nutritional RCTs with the potential to assist in prioritising those nutritional interventions which may be more likely to be effective in reducing cancer risk or progression while de-prioritising other interventions where evidence from MR suggests they are unlikely to alter subsequent disease risk or prognosis.

The challenge remains that nutritional exposures represent a complex, interconnected network of relationships—both between the exposure types themselves and with cancer. Reductionist approaches that consider a single micronutrient or macronutrient at once will only elucidate part of the relationship between diet and cancer. Recent developments in MR methodology, coupled with the growth in GWASs focusing on both granular dietary measures (e.g., micro- and macro-nutrients) and distal dietary measures (e.g., dietary patterns and preferences), may provide new opportunities to identify modifiable causal nutritional risk factors for cancer.

## Data Availability

No datasets were generated or analyzed during the current student; therefore, data sharing is not applicable to this article.

## References

[1] Doll R, Peto R (1981). The causes of cancer: quantitative estimates of avoidable risks of cancer in the United States today. JNCI.

[2] World Cancer Research Fund/American Institute for Cancer Research (2018) Diet, nutrition, physical activity and cancer: a global perspective. Continuous update project expert report

[3] Islami F (2018). Proportion and number of cancer cases and deaths attributable to potentially modifiable risk factors in the United States. CA Cancer J Clin.

[4] Blot WJ, Tarone RE (2015). Doll and Peto’s quantitative estimates of cancer risks: holding generally true for 35 years. JNCI: J Natl Cancer Inst.

[5] Parkin DM, Boyd L, Walker LC (2011). 16. The fraction of cancer attributable to lifestyle and environmental factors in the UK in 2010. Br J Cancer.

[6] Peto R, Doll R, Buckley JD, Sporn MB (1981). Can dietary beta-carotene materially reduce human cancer rates?. Nature.

[7] Die NaC (1982). National research council (US) Committee.

[8] Alpha-Tocopherol, Beta Carotene Cancer Prevention Study Group (1994) The effect of vitamin E and beta carotene on the incidence of lung cancer and other cancers in male smokers. N Engl J Med 330(15):1029–1035 10.1056/NEJM199404143301501 8127329

[9] Schatzkin A (2009). Mendelian randomization: how it can—and cannot—help confirm causal relations between nutrition and cancer. Cancer Prev Res.

[10] Smith GD, Ebrahim S (2003). 'Mendelian randomization': can genetic epidemiology contribute to understanding environmental determinants of disease?. Int J Epidemiol.

[11] Haycock PC (2016). Best (but oft-forgotten) practices: the design, analysis, and interpretation of Mendelian randomization studies. Am J Clin Nutr.

[12] Lawlor DA (2008). Mendelian randomization: using genes as instruments for making causal inferences in epidemiology. Stat Med.

[13] Davey Smith G, Hemani G (2014). Mendelian randomization: genetic anchors for causal inference in epidemiological studies. Hum Mol Genet.

[14] Smith GD (2007). Clustered environments and randomized genes: a fundamental distinction between conventional and genetic epidemiology. PLoS Med.

[15] Davey Smith G (2012). Epigenesis for epidemiologists: does evo-devo have implications for population health research and practice?. Int J Epidemiol.

[16] Swanson SA, Labrecque J, Hernán MA (2018). Causal null hypotheses of sustained treatment strategies: what can be tested with an instrumental variable?. Eur J Epidemiol.

[17] Burgess S, Labrecque JA (2018). Mendelian randomization with a binary exposure variable: interpretation and presentation of causal estimates. Eur J Epidemiol.

[18] Labrecque J, Swanson SA (2018). Understanding the assumptions underlying instrumental variable analyses: a brief review of falsification strategies and related tools. Curr Epidemiol Rep.

[19] Burgess S (2012). Use of Mendelian randomisation to assess potential benefit of clinical intervention. BMJ: Br Med J.

[20] Lawlor DA, Wade K, Borges MC, Palmer T, Hartwig FP, Hemani G, Bowden J (2019). A Mendelian Randomization dictionary: useful definitions and descriptions for undertaking, understanding and interpreting Mendelian randomization studies. OSF Prepr.

[21] Burgess S (2020). Guidelines for performing Mendelian randomization investigations [version 2; peer review: 2 approved]. Wellcome Open Res.

[22] Davey Smith G (2019). STROBE-MR: guidelines for strengthening the reporting of Mendelian randomization studies. PeerJ Prepr.

[23] Meddens SFW (2018). Genomic analysis of diet composition finds novel loci and associations with health and lifestyle. bioRxiv.

[24] Kettunen J (2016). Genome-wide study for circulating metabolites identifies 62 loci and reveals novel systemic effects of LPA. Nat Commun.

[25] Shin S-Y (2014). An atlas of genetic influences on human blood metabolites. Nat Genet.

[26] Yengo L (2018). Meta-analysis of genome-wide association studies for height and body mass index in ∼700000 individuals of European ancestry. Hum Mol Genet.

[27] Hughes DA et al (2020) Genome-wide associations of human gut microbiome variation and implications for causal inference analyses. Nat Microbiol 5:1079–1087 10.1038/s41564-020-0743-8 PMC7610462 32572223

[28] Kurilshikov A et al (2021) Large-scale association analyses identify host factors influencing human gut microbiome composition. Nat Genet 53:156–165 10.1038/s41588-020-00763-1 PMC8515199 33462485

[29] Li H (2004). A prospective study of plasma selenium levels and prostate cancer risk. J Natl Cancer Inst.

[30] Nomura AM (2000). Serum selenium and subsequent risk of prostate cancer. Cancer Epidemiol Biomark Prev.

[31] Yoshizawa K (1998). Study of prediagnostic selenium level in toenails and the risk of advanced prostate cancer. J Natl Cancer Inst.

[32] Menter DG, Sabichi AL, Lippman SM (2000). Selenium effects on prostate cell growth. Cancer Epidemiol Biomark Prev.

[33] Redman C (1998). Inhibitory effect of selenomethionine on the growth of three selected human tumor cell lines. Cancer Lett.

[34] Klein EA (2011). Vitamin E and the risk of prostate cancer: the selenium and vitamin E cancer prevention trial (SELECT). JAMA.

[35] Lippman SM (2009). Effect of selenium and vitamin E on risk of prostate cancer and other cancers: the selenium and vitamin E cancer prevention trial (SELECT). JAMA.

[36] Dennert G (1996). Selenium for preventing cancer. Cochrane Database Syst Rev.

[37] Vinceti M (2013). Friend or foe? The current epidemiologic evidence on selenium and human cancer risk. J Environ Sci Health C.

[38] Yarmolinsky J (2018). Circulating selenium and prostate cancer risk: a Mendelian randomization analysis. J Natl Cancer Inst.

[39] Mariosa D (2019). Commentary: what can Mendelian randomization tell us about causes of cancer?. Int J Epidemiol.

[40] Carreras-Torres R (2017). The role of obesity, type 2 diabetes, and metabolic factors in pancreatic cancer: a Mendelian randomization study. J Natl Cancer Inst.

[41] Gao C (2016). Mendelian randomization study of adiposity-related traits and risk of breast, ovarian, prostate, lung and colorectal cancer. Int J Epidemiol.

[42] Painter JN (2016). Genetic risk score Mendelian randomization shows that obesity measured as body mass index, but not waist: hip ratio, is causal for endometrial cancer. Cancer Epidemiol Biomark Prev.

[43] Thrift AP (2014). Obesity and risk of esophageal adenocarcinoma and Barrett's esophagus: a Mendelian randomization study. J Natl Cancer Inst.

[44] Guo Q (2017). Body mass index and breast cancer survival: a Mendelian randomization analysis. Int J Epidemiol.

[45] Cecchini RS (2016). Body mass index at diagnosis and breast cancer survival prognosis in clinical trial populations from NRG oncology/NSABP B-30, B-31, B-34, and B-38. Cancer Epidemiol Biomark Prev.

[46] Copson ER (2015). Obesity and the outcome of young breast cancer patients in the UK: the POSH study. Ann Oncol.

[47] Yin L (2013). Circulating 25-hydroxyvitamin D serum concentration and total cancer incidence and mortality: a systematic review and meta-analysis. Prev Med.

[48] Lee JE (2011). Circulating levels of vitamin D and colon and rectal cancer: the physicians' health study and a meta-analysis of prospective studies. Cancer Prev Res (Phila).

[49] Travis RC (2019). A collaborative analysis of individual participant data from 19 prospective studies assesses circulating vitamin d and prostate cancer risk. Cancer Res.

[50] Ordonez-Mena JM (2013). Serum 25-hydroxyvitamin d and cancer risk in older adults: results from a large German prospective cohort study. Cancer Epidemiol Biomark Prev.

[51] Dimitrakopoulou VI (2017). Circulating vitamin D concentration and risk of seven cancers: Mendelian randomisation study. BMJ.

[52] He Y (2018). Exploring causality in the association between circulating 25-hydroxyvitamin D and colorectal cancer risk: a large Mendelian randomisation study. BMC Med.

[53] Jiang X (2019). Circulating vitamin D concentrations and risk of breast and prostate cancer: a Mendelian randomization study. Int J Epidemiol.

[54] Yarmolinsky J (2019). Appraising the role of previously reported risk factors in epithelial ovarian cancer risk: a Mendelian randomization analysis. PLoS Med.

[55] Keum N (2019). Vitamin D supplementation and total cancer incidence and mortality: a meta-analysis of randomized controlled trials. Ann Oncol.

[56] Scragg R (2018). Monthly high-dose vitamin d supplementation and cancer risk: a post hoc analysis of the vitamin D assessment randomized clinical trial. JAMA Oncol.

[57] Afzal S (2014). Genetically low vitamin D concentrations and increased mortality: Mendelian randomisation analysis in three large cohorts. BMJ.

[58] Ong JS (2018). Vitamin D and overall cancer risk and cancer mortality: a Mendelian randomization study. Hum Mol Genet.

[59] Pilling LC (2021). Low vitamin D levels and risk of incident delirium in 351,000 older UK biobank participants. J Am Geriatr Soc.

[60] Revez JA (2020). Genome-wide association study identifies 143 loci associated with 25 hydroxyvitamin D concentration. Nat Commun.

[61] Bowden J, Davey Smith G, Haycock PC (2016). Consistent estimation in Mendelian randomization with some invalid instruments using a weighted median estimator. Genet Epidemiol.

[62] Hartwig FP, Davey Smith G, Bowden J (2017). Robust inference in summary data Mendelian randomization via the zero modal pleiotropy assumption. Int J Epidemiol.

[63] Morrison J (2020). Mendelian randomization accounting for correlated and uncorrelated pleiotropic effects using genome-wide summary statistics. Nat Genet.

[64] Burgess S, Thompson SG (2015). Multivariable Mendelian randomization: the use of pleiotropic genetic variants to estimate causal effects. Am J Epidemiol.

[65] Verbanck M (2018). Detection of widespread horizontal pleiotropy in causal relationships inferred from Mendelian randomization between complex traits and diseases. Nat Genet.

[66] Bowden J (2018). Improving the visualization, interpretation and analysis of two-sample summary data Mendelian randomization via the radial plot and radial regression. Int J Epidemiol.

[67] Hemani G, Tilling K, Davey Smith G (2017). Orienting the causal relationship between imprecisely measured traits using GWAS summary data. PLoS Genet.

[68] Locke AE (2015). Genetic studies of body mass index yield new insights for obesity biology. Nature.

[69] Cole JB, Florez JC, Hirschhorn JN (2020). Comprehensive genomic analysis of dietary habits in UK Biobank identifies hundreds of genetic associations. Nat Commun.

[70] Burgess S (2015). Using published data in Mendelian randomization: a blueprint for efficient identification of causal risk factors. Eur J Epidemiol.

[71] Yang Q (2019). Exploring and mitigating potential bias when genetic instrumental variables are associated with multiple non-exposure traits in Mendelian randomization. medRxiv.

[72] Lewis SJ, Smith GD (2005). Alcohol, ALDH2, and esophageal cancer: a meta-analysis which illustrates the potentials and limitations of a Mendelian randomization approach. Cancer Epidemiol Biomark Prev.

[73] International Agency for Research on Cancer (2012) Personal habits and indoor combustions. Volume 100 E. A review of human carcinogens. In: IARC Monogr Eval Carcinog Risks Hum, 100(Pt E):1–538 PMC4781577 23193840

[74] Cornelis MC (2016). Genome-wide association study of caffeine metabolites provides new insights to caffeine metabolism and dietary caffeine-consumption behavior. Hum Mol Genet.

[75] Davies NM, Holmes MV, Davey Smith G (2018). Reading Mendelian randomisation studies: a guide, glossary, and checklist for clinicians. BMJ.

[76] Brumpton B (2020). Avoiding dynastic, assortative mating, and population stratification biases in Mendelian randomization through within-family analyses. Nat Commun.

[77] Loh PR (2015). Efficient Bayesian mixed-model analysis increases association power in large cohorts. Nat Genet.

[78] Howe LJ (2021). Within-sibship GWAS improve estimates of direct genetic effects. bioRxiv.

[79] Davies NM (2019). Within family Mendelian randomization studies. Hum Mol Genet.

[80] Davey Smith G (2020). Mendel's laws, Mendelian randomization and causal inference in observational data: substantive and nomenclatural issues. Eur J Epidemiol.

[81] Bowden J, Davey Smith G, Burgess S (2015). Mendelian randomization with invalid instruments: effect estimation and bias detection through Egger regression. Int J Epidemiol.

[82] Zheng J (2017). Recent developments in mendelian randomization studies. Curr Epidemiol Rep.

[83] Sanderson E (2019). An examination of multivariable Mendelian randomization in the single-sample and two-sample summary data settings. Int J Epidemiol.

[84] Giambartolomei C (2014). Bayesian test for colocalisation between pairs of genetic association studies using summary statistics. PLoS Genet.

[85] Mokry LE (2015). Mendelian randomisation applied to drug development in cardiovascular disease: a review. J Med Genet.

[86] Mathers JC (2017). Nutrigenomics in the modern era. Proc Nutr Soc.

[87] Wang J (2018). Meta-analysis of human genome-microbiome association studies: the MiBioGen consortium initiative. Microbiome.

[88] Wade K, Hall L (2019). Improving causality in microbiome research: can human genetic epidemiology help? [version 1; peer review: 1 approved]. Wellcome Open Res.

[89] Saus E (2019). Microbiome and colorectal cancer: roles in carcinogenesis and clinical potential. Mol Asp Med.

[90] Consortium GT (2013). The genotype-tissue expression (GTEx) project. Nat Genet.

[91] Kettunen J (2016). Genome-wide study for circulating metabolites identifies 62 loci and reveals novel systemic effects of LPA. Nat Commun.

[92] Sun BB (2018). Genomic atlas of the human plasma proteome. Nature.

[93] Sonawane AR (2017). Understanding tissue-specific gene regulation. Cell Rep.

[94] Hekselman I, Yeger-Lotem E (2020). Mechanisms of tissue and cell-type specificity in heritable traits and diseases. Nat Rev Genet.

[95] GTEx Consortium (2020) The GTEx consortium atlas of genetic regulatory effects across human tissues. Science 369(6509):1318–1330 10.1126/science.aaz1776 PMC7737656 32913098

[96] Richardson TG (2020). A transcriptome-wide Mendelian randomization study to uncover tissue-dependent regulatory mechanisms across the human phenome. Nat Commun.

[97] Davidson LA (2004). Chemopreventive n-3 polyunsaturated fatty acids reprogram genetic signatures during colon cancer initiation and progression in the rat. Cancer Res.

[98] Evans DM (2013). Mining the human phenome using allelic scores that index biological intermediates. PLoS Genet.

[99] Evans DM, Davey Smith G (2015). Mendelian randomization: new applications in the coming age of hypothesis-free causality. Ann Rev Genom Hum Genet.

[100] Millard LAC (2015). MR-PheWAS: hypothesis prioritization among potential causal effects of body mass index on many outcomes, using Mendelian randomization. Sci Rep.

[101] Langdon RJ (2019). A phenome-wide Mendelian randomization study of pancreatic cancer using summary genetic data. Cancer Epidemiol Biomark Prev.

[102] Buniello A, MacArthur J, Cerezo M, Harris LW, Hayhurst J, Malangone C, McMahon A, Morales J, Mountjoy E, Sollis E, Suveges D, Vrousgou O, Whetzel PL, Amode R, Guillen JA, Riat HS, Trevanion SJ, Hall P, Junkins H, Flicek P, Burdett T, Hindorff LA, Cunningham F, Parkinson H (2019). The NHGRI-EBI GWAS catalog of published genome-wide association studies, targeted arrays and summary statistics. Nucleic Acids Res.

[103] Watanabe K (2019). A global overview of pleiotropy and genetic architecture in complex traits. Nat Genet.

[104] Hemani G (2018). The MR-base platform supports systematic causal inference across the human phenome. Elife.

[105] Elsworth B (2020). The MRC IEU OpenGWAS data infrastructure. bioRxiv.

[106] Kamat MA (2019). PhenoScanner V2: an expanded tool for searching human genotype-phenotype associations. Bioinformatics (Oxford, England).

[107] Millard LAC (2018). Software application profile: PHESANT: a tool for performing automated phenome scans in UK Biobank. Int J Epidemiol.

[108] Telomeres Mendelian Randomization Collaboration (2017). Association between telomere length and risk of cancer and non-neoplastic diseases: a Mendelian randomization study. JAMA Oncol.

[109] Sun YQ (2021). Assessing the role of genome-wide DNA methylation between smoking and risk of lung cancer using repeated measurements: the HUNT study. Int J Epidemiol.

[110] Burgess S (2015). Network Mendelian randomization: using genetic variants as instrumental variables to investigate mediation in causal pathways. Int J Epidemiol.

[111] Battram T (2019). Appraising the causal relevance of DNA methylation for risk of lung cancer. Int J Epidemiol.

[112] Richardson TG (2019). An integrative approach to detect epigenetic mechanisms that putatively mediate the influence of lifestyle exposures on disease susceptibility. Int J Epidemiol.

[113] Richmond RC (2018). DNA methylation as a marker for prenatal smoke exposure in adults. Int J Epidemiol.

[114] Gormley M (2020). A multivariable Mendelian randomization analysis investigating smoking and alcohol consumption in oral and oropharyngeal cancer. Nat Commun.

[115] Relton CL, Davey Smith G (2012). Two-step epigenetic Mendelian randomization: a strategy for establishing the causal role of epigenetic processes in pathways to disease. Int J Epidemiol.

[116] Carter AR (2020). Mendelian randomisation for mediation analysis: current methods and challenges for implementation. bioRxiv.

[117] Sanderson E (2020). Multivariable Mendelian randomization and mediation. Cold Spring Harb Perspect Med.

[118] Sanderson E, Spiller W, Bowden J (2020). Testing and correcting for weak and pleiotropic instruments in two-sample multivariable mendelian randomisation. bioRxiv.

[119] Holmes MV, Davey Smith G (2018). Challenges in interpreting multivariable mendelian randomization: might “good cholesterol”; be good after all?. Am J Kidney Dis.

[120] Parekh N, Zizza C (2013). Life course epidemiology in nutrition and chronic disease research: a timely discussion. Adv Nutr.

[121] Labrecque JA, Swanson SA (2019). Interpretation and potential biases of mendelian randomization estimates with time-varying exposures. Am J Epidemiol.

[122] Morris TT (2021). Interpretation of mendelian randomization using one measure of an exposure that varies over time. medRxiv.

[123] Richardson TG (2020). Use of genetic variation to separate the effects of early and later life adiposity on disease risk: Mendelian randomisation study. BMJ.

[124] Paternoster L, Tilling K, Davey Smith G (2017). Genetic epidemiology and Mendelian randomization for informing disease therapeutics: conceptual and methodological challenges. PLoS Genet.

[125] Yarmolinsky J (2018). Causal inference in cancer epidemiology: what is the role of mendelian randomization?. Cancer Epidemiol Biomark Prev.

[126] Howe LJ (2019). Association of polygenic risk scores for coronary artery disease with subsequent events amongst established cases. medRxiv.

[127] Mitchell RE, Paternoster L, Davey Smith G (2018). Mendelian randomization in case only studies: a promising approach to be applied with caution. Am J Cardiol.

[128] Cornish AJ (2020). Modifiable pathways for colorectal cancer: a Mendelian randomisation analysis. Lancet Gastroenterol Hepatol.

[129] Munafo MR (2018). Collider scope: when selection bias can substantially influence observed associations. Int J Epidemiol.

[130] Mahmoud O (2020). Slope-hunter: a robust method for index-event bias correction in genome-wide association studies of subsequent traits. bioRxiv.

[131] Gkatzionis A, Burgess S (2018). Contextualizing selection bias in Mendelian randomization: how bad is it likely to be?. Int J Epidemiol.

[132] Vansteelandt S, Dukes O, Martinussen T (2018). Survivor bias in Mendelian randomization analysis. Biostatistics.

[133] Carter AR (2019). Combined association of body mass index and alcohol consumption with biomarkers for liver injury and incidence of liver disease: a Mendelian randomization study. JAMA Netw Open.

[134] Ference BA (2017). Association of genetic variants related to CETP inhibitors and statins with lipoprotein levels and cardiovascular risk. JAMA.

[135] Ference BA (2015). Effect of naturally random allocation to lower low-density lipoprotein cholesterol on the risk of coronary heart disease mediated by polymorphisms in *NPC1L1*, *HMGCR* or both. J Am Coll Cardiol.

[136] Farmer RE (2019). Associations between measures of sarcopenic obesity and risk of cardiovascular disease and mortality: a cohort study and mendelian randomization analysis using the UK biobank. J Am Heart Assoc.

[137] North T-L (2019). Using genetic instruments to estimate interactions in mendelian randomization studies. Epidemiology.

[138] Mahamat-Saleh Y, Aune D, Schlesinger S (2020). 25-Hydroxyvitamin D status, vitamin D intake, and skin cancer risk: a systematic review and dose-response meta-analysis of prospective studies. Sci Rep.

[139] Burgess S, Davies NM, Thompson SG (2014). Instrumental variable analysis with a nonlinear exposure-outcome relationship. Epidemiology.

[140] Silverwood RJ (2014). Testing for non-linear causal effects using a binary genotype in a Mendelian randomization study: application to alcohol and cardiovascular traits. Int J Epidemiol.

[141] Rees JMB, Foley CN, Burgess S (2019). Factorial Mendelian randomization: using genetic variants to assess interactions. Int J Epidemiol.

[142] Wade KH (2018). BMI and mortality in UK biobank: revised estimates using mendelian randomization. Obesity.

[143] Sun Y-Q (2019). Body mass index and all cause mortality in HUNT and UK Biobank studies: linear and non-linear Mendelian randomisation analyses. BMJ.

[144] Staley JR, Burgess S (2017). Semiparametric methods for estimation of a nonlinear exposure-outcome relationship using instrumental variables with application to Mendelian randomization. Genet Epidemiol.

[145] Fehringer G (2016). Cross-cancer genome-wide analysis of lung, ovary, breast, prostate, and colorectal cancer reveals novel pleiotropic associations. Cancer Res.

[146] Schumacher FR (2018). Association analyses of more than 140,000 men identify 63 new prostate cancer susceptibility loci. Nat Genet.

[147] McKay JD (2017). Large-scale association analysis identifies new lung cancer susceptibility loci and heterogeneity in genetic susceptibility across histological subtypes. Nat Genet.

[148] Mills MC, Rahal C (2019). A scientometric review of genome-wide association studies. Commun Biol.

[149] Millwood IY (2019). Conventional and genetic evidence on alcohol and vascular disease aetiology: a prospective study of 500 000 men and women in China. Lancet.

[150] Clarke TK (2017). Genome-wide association study of alcohol consumption and genetic overlap with other health-related traits in UK Biobank (N=112 117). Mol Psychiatry.

[151] Schmit SL (2019). Novel common genetic susceptibility loci for colorectal cancer. J Natl Cancer Inst.

[152] Gao G (2017). Trans-ethnic predicted expression genome-wide association analysis identifies a gene for estrogen receptor-negative breast cancer. PLoS Genet.

[153] Lu Y (2019). Large-scale genome-wide association study of east asians identifies loci associated with risk for colorectal cancer. Gastroenterology.

[154] Hoffmann TJ (2015). A large multiethnic genome-wide association study of prostate cancer identifies novel risk variants and substantial ethnic differences. Cancer Discov.

[155] Shiga Y (2018). Genome-wide association study identifies seven novel susceptibility loci for primary open-angle glaucoma. Hum Mol Genet.

[156] Al Olama AA (2014). A meta-analysis of 87,040 individuals identifies 23 new susceptibility loci for prostate cancer. Nat Genet.

[157] De Vivo I (2014). Genome-wide association study of endometrial cancer in E2C2. Hum Genet.

[158] Shukla Y, Arora A (2003). Enhancing effects of mustard oil on preneoplastic hepatic foci development in Wistar rats. Hum Exp Toxicol.

[159] Dutta U (2019). Epidemiology of gallbladder cancer in India. Chin Clin Oncol.

[160] Dixit R (2013). Association of mustard oil as cooking media with carcinoma of the gallbladder. J Gastrointest Cancer.

[161] Lv J (2015). Consumption of spicy foods and total and cause specific mortality: population based cohort study. BMJ (Clinical research ed).

[162] Chen Z (2005). Cohort profile: the kadoorie study of chronic disease in china (KSCDC). Int J Epidemiol.

[163] Nagai A (2017). Overview of the BioBank Japan project: study design and profile. J Epidemiol.

[164] Vimaleswaran KS (2017). Gene–nutrient interactions on metabolic diseases: findings from the GeNuIne collaboration. Nutr Bull.

[165] Vimaleswaran KS (2020). A nutrigenetics approach to study the impact of genetic and lifestyle factors on cardiometabolic traits in various ethnic groups: findings from the GeNuIne collaboration. Proc Nutr Soc.

[166] Sandu MR, Beynon R, Richmond R, Santos Ferreira DL, Hackshaw-McGeagh L, Davey Smith G, Metcalfe C, Lane A, Martin R (2019). Two-step randomisation: applying the results of small feasibility studies of interventions to large-scale Mendelian randomisation studies to robustly infer causal effects on clinical endpoints. Preprints.

[167] Beynon RA (2019). Investigating the effects of lycopene and green tea on the metabolome of men at risk of prostate cancer: the ProDiet randomised controlled trial. Int J Cancer.

